# The impact of different doses of vitamin A supplementation on male and female mortality. A randomised trial from Guinea-Bissau

**DOI:** 10.1186/1471-2431-11-77

**Published:** 2011-09-01

**Authors:** Dorthe Yakymenko, Christine S Benn, Cesario Martins, Birgitte R Diness, Ane B Fisker, Amabelia Rodrigues, Peter Aaby

**Affiliations:** 1 Bandim Health Project, Indepth Network, Apartado 861, Bissau, Guinea-Bissau; 2 Bandim Health Project, Statens Serum Institut, Artillerivej 5, 2300 Copenhagen S Denmark; 3 Department of Infectious Diseases, Skejby Sygehus, Aarhus, Denmark

## Abstract

**Background:**

Vitamin A supplementation (VAS) given to children between 6 months and 5 years of age is known to reduce mortality in low-income countries. We have previously observed that girls benefit more from a lower dose of VAS than the one recommended by WHO, the effect being strongest if diphtheria-tetanus-pertussis vaccine (DTP) was the most recent vaccination. We aimed to test these observations.

**Methods:**

During national immunisations days in Guinea-Bissau, West Africa, combining oral polio vaccination and VAS, we randomised 8626 children between 6 months and 5 years of age to receive the dose of VAS recommended by WHO or half this dose. Mortality rate ratios (MRRs) were assessed after 6 and 12 month.

**Results:**

The overall mortality rate among participants was lower than expected. There was no significant difference in mortality at 6 months and 12 months of follow up between the low dose VAS group and the recommended dose VAS group. The MRRs were 1.23 (0.60-2.54) after 6 months and 1.17 (0.73-1.87) after 12 months. This tendency was similar in boys and girls. The low dose was not associated with lower mortality in girls if the most recent vaccine was DTP (MRR = 0.60 (0.14-2.50) after 6 months).

**Conclusion:**

Our sample size does not permit firm conclusions since mortality was lower than expected. We could not confirm a beneficial effect of a lower dose of VAS on mortality in girls.

**Trial registration:**

The study was registered under clinicaltrials.gov, number NCT00168636

## Background

Vitamin A supplementation (VAS) given to children above 6 months of age may reduce overall mortality in low-income countries by 23-30%[1-3]. The World Health Organization (WHO) currently recommends high-dose VAS at immunisation contacts after 6 months of age. Children between 6 and 12 months are advised a dose of 100,000 IU and children older than 12 months, a dose of 200,000 IU; every 4 to 6 months [4].

The reduction in mortality is assumed to be due to treatment and prevention of vitamin A deficiency (VAD)[1]. However, this may not be the only explanation. Routine vaccines during childhood have been shown to have non-specific effects on overall mortality [5-9]. While the live BCG and measles vaccine seem to confer protection against other diseases than the targeted diseases [5-8], the inactivated diphtheria-tetanus-pertussis (DTP) vaccine has been associated with increased overall mortality in girls [5,9]. Our group hypothesised that the effect of VAS on mortality may depend on an amplification of the non-specific effects of the vaccines [10]. If this is true, a high dose of vitamin A would not necessarily be better than a low dose, especially for girls who were also receiving DTP.

Previous trials have in fact indicated that a lower dose of vitamin A might be more beneficial than a higher dose in reducing mortality [11,12] and morbidity [13]. One of the first randomised vitamin A trials used small weekly doses of vitamin A instead of a high-dose supplement and that study found the strongest beneficial effect of vitamin A supplementation [14]. In a trial comparing children who received 25,000 IU or 100,000 IU of vitamin A at 9 months of age, those receiving the lower dose had significantly lower mortality by 12 months of age [11]. In 2002, our group conducted a trial during an oral polio vaccine (OPV) and VAS campaign in Guinea-Bissau, randomising children between 6 months and 5 years of age to the dose recommended by WHO and half that dose. We found that among girls the low dose was associated with a significant reduction in mortality at 6 and 9 months of follow-up [12]. This difference was mainly seen in girls 18 month of age or older [12] and in girls who had a DTP vaccine as their most recent vaccine before the enrolment in the trial (unpublished results).

We aimed to test the observations made in our former trial by testing the *a priori *hypothesis that a lower dose of vitamin A compared with the recommended dose is associated with lower mortality in girls. We furthermore hypothesised that the lower dose would be particularly beneficial in girls who had DTP vaccine at their most recent vaccination contact compared with girls who had other vaccines.

## Methods

### Setting

In Guinea-Bissau, West Africa, the Bandim Health Project (BHP) runs a health and demographic surveillance system (HDSS) in six suburban districts of the capital Bissau. There are three health centres in the study area. Guinea-Bissau has one paediatric ward, situated a few kilometres from the study area, where children are admitted in case of severe illness. During the study period from November 2004-November 2005, the mortality rate among 6-60-month-old children in the study area was 0.012 and the stunting prevalence was 16%.

Up to the age of 3 years, all children in the study area are visited by the BHP assistants every 3 months to obtain information on vital status, hospitalisations, vaccinations, infections, etc. Older children are visited approximately once per year. Information on vaccinations is also registered by the BHP at the three health centres and upon admission to the paediatric ward.

In Guinea-Bissau national immunisation days (NIDs) are conducted regularly. In 2004, all children below 5 years of age were targeted in two OPV campaigns in October and in November. In November all children between 6 months and 5 years of age were also offered VAS. The campaigns were carried out by staff from the local health centres, who went from house to house providing the treatment to target children. In the study area of the BHP, trained fieldworkers followed the campaign staff from the local health centres with a list of all children in the area generated from the HDSS database. All fieldworkers were followed by a supervisor at least one day during the campaign.

### Ethical considerations

The trial was conducted according to the guidelines laid down in the Declaration of Helsinki and all procedures involving human subjects were approved by the Central Ethical Committee in Denmark and the Ministry of Health's Committee for research in Guinea-Bissau. Verbal informed consent was obtained from the mother/guardian of all children by the staff from the health centre and recorded by the field worker from the BHP. Consent was verbal since more than 70% of the mothers are illiterate. The purpose of the study was clearly explained and by drawing a lot it was made clear that they took part in a randomised study.

### Enrolment and randomisation to two different doses of VAS

Children between 6 months and 5 years of age residing in the BHP study area in November 2004 were eligible for enrolment in the present trial. Exclusion criteria were 1) signs of clinical vitamin A deficiency, and 2) VAS within the last month prior to the date of visit.

Randomisation envelopes were prepared prior to the campaign. The envelopes contained 100 lots, 50 marked "WHO" and 50 marked "BHP". The mothers were explained that vitamin A was given because it reduces morbidity and mortality, but that there is no clear evidence which dose is the best. If the mother agreed to participate she drew a lot from the envelope indicating which dose of vitamin A her child should receive. If the lot said "WHO", the child would receive 100,000 IU if it was younger than 1 year of age, and 200,000 IU if it was 1 year or older. If the lot said "BHP" the dose would be 50,000 IU if the child was younger than 1 year of age, and 100,000 IU if the child was 1 year or older. If the mother did not want her child to participate, the child was given the dose recommended by the WHO. The vitamin A was distributed in gelatine capsules containing retinyl palmitate (International Dispensary Association, The Netherlands).

The field workers and the participants were not blinded to the randomisation. The child's vaccination card was inspected, existing information on vaccine status was verified, and new vaccines noted on the list.

### Outcomes

The main outcome was mortality after 6 and 12 months of follow-up.

Trained fieldworkers visited all enrolled children after 6 months. There was no information about the allocation on the follow-up forms. Vital status was confirmed, the child's vaccination card was inspected, and new vaccines were registered. If the vaccination card was not seen, information on vaccinations status was obtained from the HDSS. The data from the campaign and from the 6-month visit were double entered and controlled by comparing the data with the information from the HDSS.

Information on survival and vaccination status after 12 months of follow-up was obtained during the NIDs in November 2005. We controlled the data entry by double-entering 10% of the data, and found a 99.1% degree of consistency. Furthermore, information on vital status and dates of vaccinations was compared with information from the HDSS. In case of inconsistency, information was checked by visiting the household again.

If a child had died, a local doctor interviewed the mother or guardian using a verbal autopsy questionnaire [15]. A panel of three medical doctors, including at least one local doctor, all blinded to the randomisation group, reached consensus on the cause of death.

### Participation in a neonatal VAS trial

In the period from August 2002 to November 2004, the BHP conducted a trial randomising children to receive vitamin A or placebo at birth together with BCG (the "Vitamin-A-at-birth" trial)[16]. We linked our data to the "Vitamin-A-at-birth" trial, also including children from the preceding pilot study, which took place from August 2002 to November 2002 to study whether VAS at birth modified the response to the different doses [17].

### Non-participants

**Figure 1:**
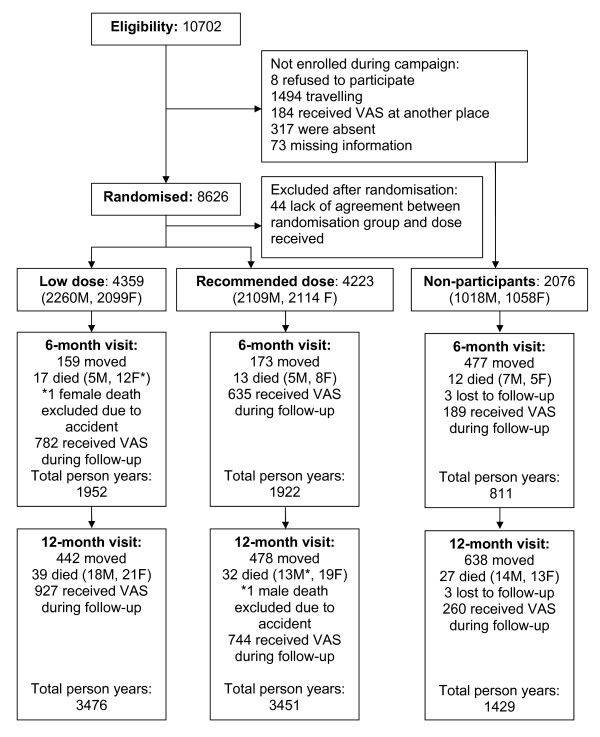
**Flow chart**.

We compared trial participants with the children who were eligible for enrolment, but were travelling during the campaign or not participating for other reasons (Figure 1). We did not have a 6-month follow-up visit to obtain information on survival and vaccination, but used the HDSS data. This approach resulted in fewer children in the older age groups because the HDSS only visits children up to 3 years of age.

### Samples size considerations

We aimed to recruit all eligible children in our study area, hence including two more districts than in our previous trial. We expected to be able to recruit approximately 7500 children. With an expected mortality of 1.2% at 6 months of follow-up it would be possible to detect a 55% reduction in mortality among girls who received the low dose compared with the recommended dose within the first year with a power of 80% and a two-sided significance level of 0.05. In the previous trial we had found the low dose to be associated with a 72% reduction in female mortality after 9 months [12].

### Data analysis and statistical methods

Statistical analysis was conducted using Stata/SE 9.2 (Stata corporation, Texas, USA). The analyst was not blinded to randomisation group while doing the analyses. Baseline characteristics were compared by chi^2-tests and by t-tests. Mortality rate ratios (MRRs) were computed in Cox proportional hazards models with time since enrolment as the underlying time. Children who died due to accidents were censored from the analyses at the time of the death. We tested for confounding on the outcome death within 12 months of follow-up by including background variables in the model one by one, defining confounders as variables which changed the estimate by more than 5%. In all analyses children were censored if they received VAS during follow-up (Figure 1). When analysing the effect of most recent vaccination before the campaign, we censored children when they received a vaccine during follow-up. As the date of vaccination, we used the date of registration instead of the actual day of vaccination to avoid survival bias [18].

## Results

### Baseline characteristics

A total of 10702 children were eligible for randomisation. No children were excluded due to clinical signs of VAD. Of the eligible children 8626 (80.6%) were enrolled in the trial between November 18-21 2004. The main reason for not being enrolled was travelling (1494 (14.0%)) of all eligible children) (Figure 1). Due to conflicting information between dose and age, 44 (0.5%) children were excluded. A total of 4223 (49.2%) received the WHO recommended dose and 4359 (50.8%) received the low dose. The randomisation groups were comparable with regard to sex, vaccination status and socio-economic characteristics (Table 1). However, the groups differed with regards to age distribution with more children between 6 and 11 months of age being in the low-dose group. As a consequence more children in the low-dose group had also been enrolled in the "Vitamin-A-at-birth" trial (Table 1). Adjusting for VAS-at-birth did not change the estimate on overall mortality by more than 5%, and final estimates were therefore only adjusted for age.

**Table 1:** Baseline characteristics

	Vitamin A Low dose (%)	Vitamin A High dose (%)	P-value *Low doses vs. High dose*	Non-participants (%)	P-value *Non-participants vs. Participants*
**Number**	4359	4223		2076	

**Sex**					

Male	2260 (51.8)	2109 (50.0)		1018 (49.0)	

Female	2099 (49.2)	2114 (50.0)	0.08	1058 (51.0)	0.13

**Age (months)**					

6-11	661 (15.2)	513 (12.1)		330 (15.9)	

12-17	510 (11.7)	478 (11.3)		289 (13.9)	

18-35	1464 (33.6)	1463 (34.6)		677 (34.1)	

36-60	1724 (39.5)	1769 (41.9)	< 0.001	780 (37.6)	< 0.001

**Type of most recent vaccine^1^**					

BCG/MV	1565 (35.9)	1492 (35.3)		704 (33.8)	

DTP	2240 (51.4)	2186 (51.7)	0.94	988 (47.7)	< 0.001

**"Vitamin-A-at-birth" trial**					

VAS-at-birth	634 (50.7)	514 (49.2)		250 (45.6)	

Placebo	617 (49.3)	530 (50.8)	0.04	298 (54.4)	0.06

**District**					

Bandim	1927 (44.2)	1834 (43.4)		1018 (49.0)	

Belem/Mindara	800 (18.3)	746 (17.7)		356 (17.2)	

Cuntum	1632 (37.4)	1643 (38.9)	0.36	702 (33.8)	< 0.001

**Ethnic group**					

Pepel	1359 (30.2)	1289 (30.5)		603 (29.0)	

Fula	634 (14.5)	623 (14.7)		404 (19.5)	

Manjaco	553 (12.7)	569 (13.5)		228 (11.0)	

Others	1813 (41.6)	1742 (41.3)	0.70	841 (40.5)	< 0.001

**Maternal education^3 ^(years)**					

0	1318 (30.2)	1255 (29.7)		800 (38.5)	

1-4	706 (16.2)	707 (16.7)		345 (16.6)	

5-6	699 (16.0)	654 (15.5)		269 (13.0)	

≥ 7	1160 (26.6)	1147 (27.2)	0.87	396 (19.1)	< 0.001

**Maternal age^3 ^(years)**					

≤ 18	194 (4.5)	153 (3.6)		87 (4.2)	

19-24	1237 (28.4)	1196 (28.3)		645 (31.1)	

25-29	1168 (26.8)	1192 (28.2)		617 (29.7)	

≥ 30	1366 (31.3)	1320 (31.3)	0.22	526 (25.3)	< 0.001

**Number of siblings^3^**					

0-1	1399 (32.1)	1387 (32.8)		720 (34.7)	

2-3	1703 (39.1)	1682 (39.8)		879 (42.3)	

≥ 4	1068 (24.5)	986 (23.3)	0.48	414 (19.9)	< 0.001

**Type of roof^2^**					

Straw	249 (5.7)	246 (5.8)		120 (5.8)	

Zinc (hard)	3919 (89.9)	3803 (90.0)	0.85	1890 (91.0)	0.07

**Electricity^2^**					

No	2926 (67.1)	2868 (67.9)		1489 (71.7)	

Yes	1240 (28.5)	1182 (28.0)	0.66	521 (25.1)	< 0.001

^1^Of all participating and non-participating, 763 children received DTP+MV together, 720 not vaccinated or no information

^2^Information at time of childbirth, numbers do not add up due to missing information

### Main analyses of mortality

Information on survival was obtained for all children at 6 months and 12 months of follow-up. A total of 73 children died within 12 months of follow-up. For two children the death was due to an accident (1 male in the high-dose group, and 1 female in the low-dose group), and they were therefore censored in the analysis at the time of death. The overall mortality rate (MR) after 12 months of follow-up was 0.010 (71 deaths/6927 person years at risk).

There was no significant difference in mortality at 6 months and 12 months of follow up between the low dose VAS group and the recommended dose VAS group; the MRR being 1.23 (0.60-2.54) after 6 months and 1.17 (0.73-1.87) after 12 months. The MRRs did not differ between boys and girls (Table 2).

**Table 2:** Mortality after 6 and 12 months of follow-up in children who received a lower dose compared with the recommended dose of vitamin A, Guinea-Bissau 2004-2005

	All (n = 8582)	Boys (n = 4369)	Girls (n = 4213)
**At 6 months of follow-up**	Mortality Rate (deaths/years at risk)		

Low dose	0.009 (17/1952)	0.005 (5/1005)	0.013 (12/947)

High dose	0.007 (13/1922)	0.005 (5/955)	0.008 (8/968)

Mortality Rate Ratio^1 ^(95% CI)	1.23 (0.60-2.54)	0.92 (0.27-3.17)	1.43 (0.59-3.51)

P for same effect in boys and girls		0.57	

**At 12 months of follow-up**	Mortality Rate (deaths/years at risk)		

Low dose	0.011 (39/3476)	0.010 (18/1788)	0.012 (21/1688)

High dose	0.009 (32/3451)	0.008 (13/1714)	0.011 (19/1737)

Mortality Rate Ratio^1 ^(95% CI)	1.17 (0.73-1.87)	1.30 (0.64-2.65)	1.08 (0.58-2.02)

P for same effect in boy and girls		0.71	

^1 ^Adjusted for sex and age as a continuous variable

The low dose of VAS in girls was not significantly different depending on the most recent vaccination. After 6 months of follow-up, the MRR was 0.60 (0.14-2.50) if the most recent vaccination was DTP and 3.06 (0.63-15) if the most recent vaccine was BCG vaccine or measles vaccine (p for same effect = 0.12). After 12 months the estimates were 0.77 (0.29-2.07) and 1.21 (0.45-3.26) respectively (p for same effect 0.55).

### Causes of death

The cause of death was determined by verbal autopsy in 56 (77%) of the 73 dead children. Information on cause of death from a brief interview made shortly after death was available for 8 of the remaining 17 children. There were no significant difference in cause of death between the two randomisation groups (results not shown), but there were too few deaths to allow for a meaningful comparison; hence, the data mainly served to excluded deaths due to accidents.

### Post hoc analyses

There was no evidence that a lower dose was more beneficial in girls above 18 months of age (Table 3). Previous studies have suggested limited beneficial effect of micronutrient supplementation in girls below 12 months of age [19]. In this study there was a tendency for the lower dose being more beneficial in the youngest children (p = 0.06 for same effect in children below and above 12 months of age) which was not limited to girls (Table 3).

**Table 3:** Mortality after 12 months of follow-up comparing a lower dose of vitamin A with the recommended dose among subgroups

	Number of deaths/years at risk		Mortality Rate Ratio^1 ^(95% CI) (Low dose vs. Recommended dose)		
	**Low dose**	**Recommended dose**			

	**All** **(n = 8582)**		**All** **(n = 8582)**	**Boys** **(n = 4369)**	**Girls** **(n = 4213)**

***Age groups***					

6-11 months	7/397	11/327	0.54 (0.21-1.38)	0.39 (0.07-2.11)	0.63 (0.20-1.99)

12-17 months	7/316	4/301	1.65 (0.48-5.63)	1.34 (0.30-6.00)	2.73 (0.28-26)

18-60 months	25/2763	17/2823	1.50 (0.81-2.77)	1.97 (0.74-5.25)	1.22 (0.54-2.72)

P for same effect in age groups			0.17	0.27	0.46

***VAS at birth***					

	**All** (n = 2295)		**All** (n = 2295)	**Boys** (n = 1182)	**Girls** (n = 1113)

VAS at birth	5/308	3/260	1.30 (0.31-5.46)	0.89 (0.05-15)	1.50 (0.27-8.18)

Placebo at birth	2/289	6/251	0.29 (0.06-1.41)	0.79 (0.05-13)	0.18 (0.02-1.52)

P for same effect by VAS at birth			0.17	0.93	0.12

^1 ^Adjusted for sex and age as a continuous variable.

We have recently observed that the mortality effect of VAS at 12 months of age was significantly different in females who had received VAS or placebo at birth [17]. In the present trial the response to the different doses did not depend significantly on whether a child had received VAS at birth (Table 3).

### Comparison of participants and non-participants

There were notable differences in baseline characteristics between participants and non-participants (Table 1). Adjusting for age and type of most recent vaccine changed the estimate on overall mortality by more than 5%, and all comparisons between participants and non-participants were therefore adjusted for these variables.

**Figure 2:**
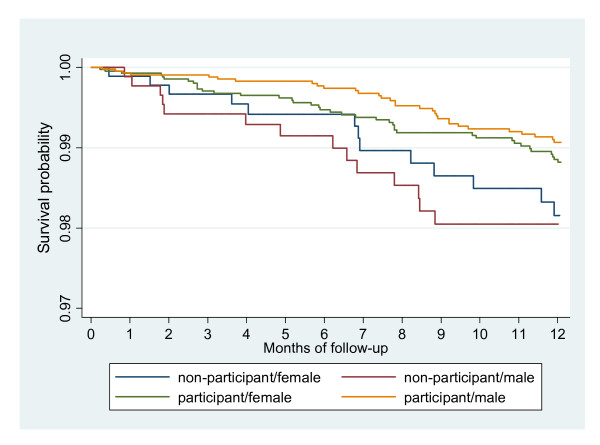
**Kaplan-Meier survival curves in boys and girls, comparing participants with non-participants**.

Among the non-participants 2073 (99.9%) children could be followed for 1429 person years during 12 months. In this group 27 children died (MR = 0.019), none of them due to accident (Figure 1). We found a borderline significant reduction in mortality for those who participated in the trial (MRR after 12 months of follow-up: 0.64 (0.41-1.01)). For boys this reduction was consistent and significant both after 6 months (MRR = 0.36 (0.13-0.94)) and 12 months of follow-up (MRR = 0.52 (0.28-0.99)), whereas for girls there was no significant effect of participating, the MRRs after 6 and 12 months being 1.05 (0.39-2.84) and 0.77 (0.41-1.45), respectively (p = 0.11 and p = 0.10 for same effect in boys and girls after 6 and 12 months of follow-up, respectively)(Figure 2).

## Discussion

In our previous trial, the low dose of VAS was associated with an overall MRR of 0.69 (0.36-1.35), based on a strong beneficial effect in females (0.19 (0.06-0.66) after 6 months of follow-up; 0.28 (0.11-0.70) after 9 months)[12]. This effect was particularly seen among the older girls and girls who had DTP as their last vaccine. In the present trial, which was designed to test this observation, we were not able to confirm the beneficial effect of a lower dose of VAS on overall female mortality, nor did we confirm a significant beneficial effect of the low dose among girls with DTP as their most recent vaccine before enrolment. If anything, the youngest children below 12 months of age benefitted more from receiving a lower dose. Compared with non-participants, it was only boys, who benefited significantly from participating in the campaign.

### Strengths and weaknesses

Strengths of the present trial include the randomised design and the large number of participants.

Weaknesses include that the two randomisation groups differed in regard to age with an overweight of younger children the low-dose group. This pattern was consistent within the whole study area, and since the fieldworkers were under supervision during the campaign this is most likely a chance event. We controlled all analyses for age, but cannot exclude that there are other underlying differences between the two groups which were not adjusted for.

As expected the non-participants differed significantly from participants with regard to many baseline characteristics, indicating strong selection bias. We have attempted to control for all known confounders, but the comparisons between participants and non-participants should nonetheless be interpreted with great caution. Selection bias, however, is unlikely to explain why only boys benefitted significantly from participation in the study.

The trial was not blinded, but this is not likely to affect an outcome like mortality.

### Potential explanations for contrasting results

We explored the potential explanations for the lack of consistency between our previous trial, which found a strong beneficial effect of a low dose in girls, and the present trial. Though our findings were based on small numbers and not significant, *post hoc *analyses showed a similar beneficial effect of the low dose in girls who had been randomised to placebo at birth. In contrast, girls who had received VAS at birth seemed to benefit more from the higher dose. That neonatal VAS primes the response to a subsequent dose of VAS is supported by a recent observation within the "vitamin-A-at-birth"-trial. In that trial girls who had received VAS at birth benefitted significantly from receiving a high dose of vitamin A at age 12 months compared with girls who had received placebo at birth [17].

This could be a possible explanation for the divergent results in the present trial and the previous 2002 trial; in 2002 no children had received VAS at birth.

### Sex-differences in the response to VAS

Many studies which analysed data by sex have found sex-differences in the response to VAS [12,19-25]. Overall sex-differences have not been confirmed in meta-analyses [1-3], but it should be noted that the analyses by sex have been based on a limited number of trials, since surprisingly many have not reported data by sex, and furthermore the meta-analyses have not taken vaccination status into account. The present trial did not clearly confirm previous observations of sex-differences in response to VAS. However, though the results were by no way significant, as seen in the previous trial, girls tended to benefit more from a lower dose of VAS if DTP was the most recent vaccine than if the most recent vaccine was BCG or measles vaccine. Furthermore, only boys benefitted significantly from participating in the campaign. These sex-difference may be due to a negative interaction between VAS and DTP in girls [23-25].

### Context

Our trial was done in a setting with a low prevalence of clinical VAD. None of the 8626 children were excluded from the trial due to clinical VAD. At 4 months of age 16% of the children in the study area had low levels of retinol binding protein indicating VAD [26], and that figure was 9% when controlled for CRP [26]. The impact of VAS may be different in settings with a higher prevalence of VAD.

## Conclusions

In conclusion, we did not confirm the previously observed effect of a lower dose of vitamin A being more beneficial for girls at either 6 or 12 months of follow-up.

## Competing interests

The authors declare that they have no competing interests.

## Authors' contributions

DY was the chief investigator and is the guarantor. DY, CSB, CM, AR and PA designed the study. DY, CM, BRD, ABF and PA initiated the trial and supervised the data collection during the campaign. DY was responsible for the statistical analysis with assistance from CSB. DY wrote the first draft of the paper. All authors contributed to and approved the final version of the paper.

## Pre-publication history

The pre-publication history for this paper can be accessed here:

http://www.biomedcentral.com/1471-2431/11/77/prepub
